# Coenzyme Q at the Hinge of Health and Metabolic Diseases

**DOI:** 10.3390/antiox10111785

**Published:** 2021-11-08

**Authors:** Juan Diego Hernández-Camacho, Laura García-Corzo, Daniel José Moreno Fernández-Ayala, Plácido Navas, Guillermo López-Lluch

**Affiliations:** Centro Andaluz de Biología del Desarrollo, Instituto de Salud Carlos III, Universidad Pablo de Olavide-CSIC-JA, and CIBERER, 41013 Sevilla, Spain; jdhercam@upo.es (J.D.H.-C.); lgarcor@upo.es (L.G.-C.); dmorfer@upo.es (D.J.M.F.-A.); pnavas@upo.es (P.N.)

**Keywords:** coenzyme Q, ubiquinone, metabolic disease, rare disease, mitochondria

## Abstract

Coenzyme Q is a unique lipidic molecule highly conserved in evolution and essential to maintaining aerobic metabolism. It is endogenously synthesized in all cells by a very complex pathway involving a group of nuclear genes that share high homology among species. This pathway is tightly regulated at transcription and translation, but also by environment and energy requirements. Here, we review how coenzyme Q reacts within mitochondria to promote ATP synthesis and also integrates a plethora of metabolic pathways and regulates mitochondrial oxidative stress. Coenzyme Q is also located in all cellular membranes and plasma lipoproteins in which it exerts antioxidant function, and its reaction with different extramitochondrial oxidoreductases contributes to regulate the cellular redox homeostasis and cytosolic oxidative stress, providing a key factor in controlling various apoptosis mechanisms. Coenzyme Q levels can be decreased in humans by defects in the biosynthesis pathway or by mitochondrial or cytosolic dysfunctions, leading to a highly heterogeneous group of mitochondrial diseases included in the coenzyme Q deficiency syndrome. We also review the importance of coenzyme Q levels and its reactions involved in aging and age-associated metabolic disorders, and how the strategy of its supplementation has had benefits for combating these diseases and for physical performance in aging.

## 1. Introduction

Coenzyme Q (CoQ) or ubiquinone is an essential element for mitochondrial function and provides antioxidant protection of cell membranes and plasma lipids [1]. CoQ was isolated and identified by Festenstein et al. in 1955 [2], and validated by Crane et al. in 1957, who demonstrated its main function in the mitochondrial electron transport chain (ETC) [3]. CoQ also shows a potent antioxidant function in plasma membranes, endomembranes, and lipopoteins [4].

The structure of CoQ is essentially the same in all organisms, composed of a benzoquinone ring bound to a polyisoprenoid side chain that preserve the molecule inserted into a lipid bilayer. Isoprene units of the chain are species-specific: human isoform mainly encloses ten isoprene units (CoQ_10_). In rodents, this molecule presents mainly nine units (CoQ_9_), and around a 10% has ten units (CoQ_10_). The yeast *Saccharomyces cerevisiae* holds six units (CoQ_6_), whereas *Schizosaccharomyces pombe* contains ten units (CoQ_10_). Bacteria such as *Escherichia coli* contains eight isoprene units (CoQ_8_) [5]. 

Regarding its redox activity, CoQ can be found in a reduced form (CoQH_2_ or ubiquinol), in a completely oxidized state (CoQ or ubiquinone), or in a semi-reduced state known as a semi-ubiquinone (CoQH^•^). Through this redox cycle, CoQ plays an essential role in the ETC maintaining the electron flux from complexes I and II to complex III, leading a chemiosmotic gradient that is used by the ATP synthase to produce ATP by oxidative phosphorylation (OXPHOS) [6]. 

The CoQ-dependent oxidation/reduction cycle also confers to this molecule the property to transfer electrons in different biological processes and to act as an antioxidant at cell membranes preserving lipids, protein, and nucleic acids from oxidative damage [7,8]. By this mechanism, CoQ is the major antioxidant preventing lipid peroxidation in cell membranes and lipoproteins [9,10]. Further, CoQ also plays a key role in the maintenance of vitamin C and α-tocopherol antioxidant activities at the plasma membrane, leading the regulation of cellular redox homeostasis [11,12]. 

CoQ is involved in essential physiological processes in cells and tissues, and its homeostasis is maintained through highly conserved regulatory mechanisms of the biosynthesis pathway [13]. Defects in either the biosynthesis pathway or in the mitochondrial functions that affect CoQ homeostasis in tissues and organs imply an activation of metabolic diseases and aging, in which mitochondrial dysfunction is involved [1,14]. This review highlights the importance of CoQ in the maintenance of health and in the evolution of metabolic diseases and aging. 

## 2. Biosynthesis of CoQ

**Figure 1:**
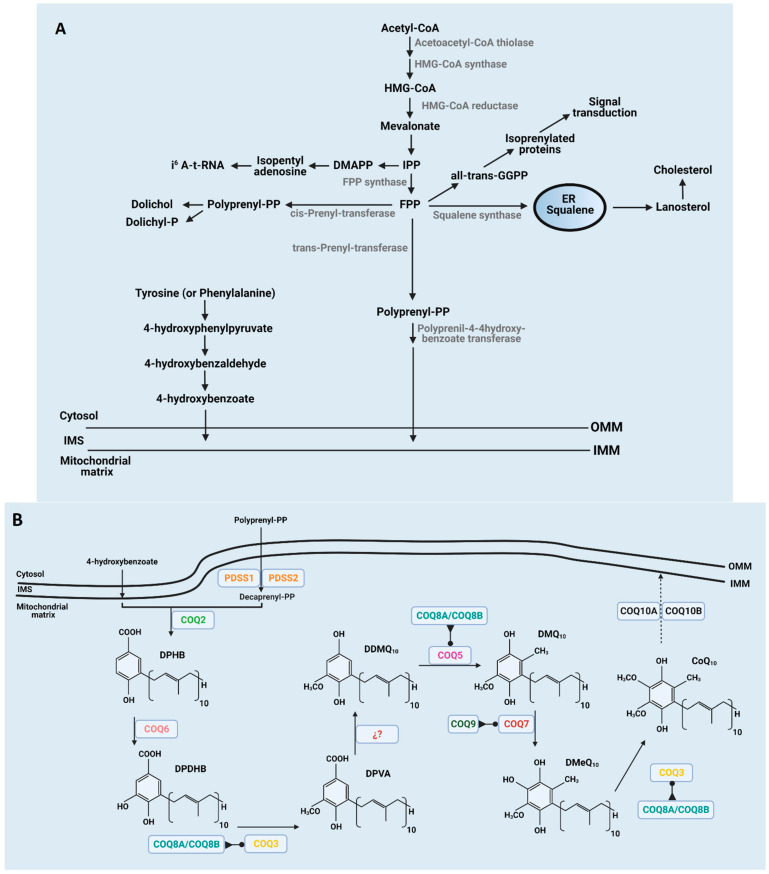
Scheme of CoQ synthesis in mitochondria. (**A**) Synthesis of the mevalonate chain in the ER and quinone head processing from tyrosine in cytosol or mitochondrial matrix. (**B**) Assembly of benzene ring and isoprenoid tail at the inner mitochondrial membrane and further modifications of the phenol head at the inner mitochondrial membrane. Members of the CoQ-synthome are indicated in each step. In some cases, the enzyme involved in some of the steps are unknown. Regulatory enzymes such as COQ8 and COQ9 are indicated at the steps in which they are actively involved.

Although CoQ can be incorporated through the diet, most of the CoQ is endogenously produced inside the mitochondria of every cell and then distributed throughout the cellular membranes [15]. An overview of the CoQ biosynthesis pathway in mammals is depicted in Figure 1. The biosynthesis pathway, in which at least 13 genes are involved [16,17,18], is not fully understood, and most of the recent discoveries in humans comes from the study of CoQ deficiency syndrome [19]. Final reactions of CoQ biosynthesis take place inside mitochondria by a nuclear-encoded COQ proteins cluster, after the isoprenoid tail and benzoquinone head are bound, producing decaprenyl hydroxybenzoic acid (DPHB) at the matrix side of the inner mitochondrial membrane (IMM). A CoQ polyisoprenoid chain is produced through the mevalonate pathway in the endoplasmic reticulum shared with the synthesis of other lipids, such as cholesterol or dolichol, and heme A and protein prenylation [5]. It is initiated by the condensation of three acetyl-Coenzyme A (acetyl-CoA) molecules to form 3-hydroxy-3-methyl-glutaryl-coenzyme A (HMG-CoA) due to acetoacetyl-CoA thiolase and HMG-CoA synthase activities. Mevalonate is then produced from HMG-CoA by HMG-CoA reductase. Mevalonate is phosphorylated and decarboxylated to produce isopentenyl pyrophosphate (IPP). Farnesyl pyrophosphate synthase uses IPP to make farnesyl pyrophosphate (FPP), an intermediary product for geranyl pyrophosphate (GPP) production. The next enzyme involved in CoQ biosynthesis is transprenyl-transferase that converts FPP into polyprenyl-PP, which is considered a rate-limiting step [6]. The transport of FPP into mitochondria is not fully understood yet, but it has been proposed to occur in the ER-mitochondria contacts through the ERMES complex [17,20,21]. COQ1 (PDSS1 and PDSS2) would assemble the side chain producing decaprenyl-PP (DPP) in those species containing CoQ_10_ [16,22]. The ring precursor *para*-hydroxybenzoate (*p*HB) seems to be synthesized in cytosol including seven proteins, such as Hfd1p and its human homolog ALDH3A1 [23,24]. Recently, it has been shown in yeast that the five aminotransferases, Aro8, Aro9, Bat2, Bna3, and Aat2, are required to produce the benzoquinone ring for CoQ synthesis, which would also be studied in undiagnosed CoQ deficiency syndrome [25].

The next set of reactions occur through the interaction of *COQ* gene-encoded proteins that provide support for the existence of a CoQ biosynthesis complex or CoQ synthome [17,26]. In these reactions, a sequential series of modifications of the aromatic ring are performed. Initially, DPHB suffers from C5-hydroxylation by COQ6, resulting in decaprenyl-dihydroxybenzoate (DPDHB) formation [27]. Subsequently, DPDHB is O-methylated by COQ3 to form decaprenyl-vanillic acid (DPVA) [28,29]. Later, a C1-hydroxylation and a C1-decarboxylation are catalyzed by unidentified proteins to form demethoxy-demethyl-coenzyme Q (DDMQ). Afterwards, COQ5 carries a C2-methylation to form demethoxy-coenzyme Q (DMQ) [30]. Thereafter, DMQ is C6-hydroxylated by COQ7 to form demethyl-coenzyme Q (DMQ) [31]. Finally, COQ3 catalyzes the final step ending in CoQ from DMQ.

In recent years, other COQ proteins showing regulatory functions have been discovered. COQ8A (ADCK3) and COQ8B (ADCK4) show properties of atypical kinases that phosphorylate COQ3, COQ5, and COQ7 [32,33,34]. The function of COQ4 has not yet been elucidated, but some reports indicate that this enzyme modulates the formation and maintenance of the CoQ biosynthetic complex [35]. Moreover, COQ9 is a lipid-binding protein that modulates the activity of COQ7 [36,37]. Further, COQ10A and COQ10B possibly regulate the distribution and/or complex III binding of CoQ within the IMM where displays its functions [38]. 

## 3. CoQ as Metabolic Integrative Factor for Cellular Homeostasis

The main function of CoQ is to act as an electron carrier in the ETC that drives all electrons to complex III. Most of the redaction reactions of CoQ come from NADH-dependent complex I and FADH_2_-dependent complex II. However, CoQ is also reduced by diverse oxidoreductases, which represents key steps in important metabolic pathways, such as iron sulfur cluster synthesis, nucleotide synthesis, and sulfide metabolism [39,40]. These reactions and their metabolic mechanisms have been recently reviewed by [41].

As a ubiquitous component of eukaryotic lipid membranes, CoQ participates in electron transfer activities. In these redox activities, the redox state of CoQ determines where and how electrons move from the electron carrier and if leaks produce reactive oxygen species (ROS) [42]. Although free radicals have long been thought to be responsible for aging and age-related diseases, recent reports indicate that they have both positive and negative effects on longevity and health acting as integrators of cellular homeostasis [39,42,43,44]. The levels of CoQ, its redox state, and its interaction with CoQ-dependent enzymatic activities can be key in the modulation of cellular homeostasis. 

### 3.1. Mitochondrial Redox Reactions

#### 3.1.1. Redox Reactions at the ETC

The CoQ-dependent redox reactions in the mitochondria are linked directly or indirectly to oxidative phosphorylation (OXPHOS), in which electrons flow through mitochondrial complexes to generate the proton gradient necessary for ATP synthesis, until they finally reach oxygen to reduce it to water [39], additionally affecting diverse metabolic pathways [41].

To access ETC, the energy of the electron flows clusters the mitochondrial redox reactions into three groups, depending on their redox potential [45]. The more energetic dehydrogenases operate at approximately −280 mV and transfer electrons from metabolites to NAD^+^, reducing it to NADH, and oxidizing the proper metabolite to another with lower potential energy. Within this group, enzymes, for amino acid metabolism, such as 2-oxoadipate dehydrogenase for lysine catabolism and branched-chain dehydrogenases for leucine, isoleucine and valine catabolism; hydroxyacyl-CoA-dehydrogenase for fatty-acid β-oxidation; pyruvate dehydrogenase for carbohydrate catabolism; and the three NAD-dehydrogenases integrated in the Krebs cycle: isocitrate dehydrogenase, 2-oxoglutarate dehydrogenase, and malate dehydrogenase are included. All these dehydrogenases produce NADH to feed mitochondrial complex I, which uses the energy to generate the proton gradient necessary for ATP synthesis and transfers the electrons to ubiquinone reducing it to ubiquinol, which is the substrate for complex III.

The second group of dehydrogenases operate at a lower redox potential of approximately +20 mV. They use FAD, a flavin-adenine dinucleotide that is reduced to FADH_2_, and transfer electrons from metabolites directly to ubiquinone, without contribute to the proton gradient generation. This group includes succinate dehydrogenase (complex II), enzymes for amino acid metabolism, such as proline dehydrogenase, dihydroorotate dehydrogenase for pyrimidine nucleotide metabolism, acyl-CoA dehydrogenases for fatty-acid beta oxidation, and glycerol-3-phosphate dehydrogenase, which shuttles the glycolytic electrons from NADH directly to the mitochondrial respiratory chain to regenerate NAD^+^ and avoid the blockage of the glycolysis. All these dehydrogenases use as substrate CoQ producing ubiquinol, which is reoxidized by complex III, using this energy to generate the mitochondrial proton gradient, and finally transferring the electrons to molecules of cytochrome c [41].

The third group of dehydrogenases operate at a very low redox potential of approximately +320 mV. Here, the cytochrome c linked enzymes are included, such as the sulfite dehydrogenase necessary for cysteine catabolism, which oxidizes sulfite to sulfate and reduces cytochrome c. Finally, electrons from cytochrome c go through complex IV, contributing to the proton gradient, until they reach oxygen at +600 mV.

#### 3.1.2. CoQ Role in Reactive Oxygen Species Balance

Eleven sites in the ETC that leak electrons to oxygen producing ROS have been associated with substrate oxidation and oxidative phosphorylation in mitochondria [46]. These sites are located inside the oxoacid dehydrogenases that feed electrons to NAD^+^, but higher production has been recorded in proper mitochondrial complexes III, I, and II in the sites where CoQ presents its redox activity [45]. In addition, the rate of ROS production can vary depending on the tissue, showing higher production in brown adipose tissue compared with skeletal muscle, and being heart the organ with lower rates [47]. The type of substrate is also crucial, succinate is the metabolite that causes higher production of ROS, and both fatty acids and amino acids the metabolites that produces the lower levels [46]. Most of the hydrogen peroxide production takes place in complex I, while glycerol-3-phophate, fatty acids and glutamate/malate generate an equal amount in complexes I and III, and inside the proper glycerol-3-phosphate dehydrogenase. ROS is also produced by 2-oxoglutarate dehydrogenase.

Due to the topology of the mitochondria and the presence of a double membrane, these enzymes can generate superoxide either in the intermembrane space or in the matrix, but hydrogen peroxide exclusively in the matrix. The specific site of ROS production is important for redox signaling without generating catastrophic energetic effects [45]. Thus, we can distinguish intramitochondrial redox signaling, in which all targets are located inside mitochondria, and redox signaling from mitochondria to the rest of the cell (retrograde redox signaling). This ROS-dependent signaling process can even affect targets located outside cells. Among the targets affected by mitochondrial ROS are the initiation of hypoxia-inducible factor (HIF) signaling and its effect in gene expression modulation, insulin secretion stimulated by redox signaling due to the metabolism of branched-chain keto acids and fatty acids, retrograde redox signaling that modulates PGC1α during exercise in skeletal muscle, and the redox signaling that affects immune cells [48,49].

#### 3.1.3. Mitochondrial CoQ Is Essential for Many Different Metabolic Processes

In addition to its known activity as a redox carrier among complexes I, II, and III of the ETC, CoQ also receives electrons from many different dehydrogenases [41,50,51]. CoQ receives electrons from mitochondrial glycerol-3-phosphate dehydrogenase (G3PDH) that connects glycolysis, OXPHOS, and fatty acid metabolism [52]. CoQ is also reduced by the electron-transport flavoprotein dehydrogenase (ETFDH), an essential enzyme in β-oxidation of fatty acids and in the oxidation of branched amino acids [53]. The capacity as an electron acceptor of CoQ is also essential for the activity of proline dehydrogenase (PROD), involved in glyoxylate metabolism [54], and sulphide-quinone oxidoreductase (SQR) that participates in sulphide detoxification [55], an important regulator of many cellular processes [56,57]. Further, CoQ is also reduced by choline dehydrogenase (CHDH) [58], from choline to glycine conversion, and dihydroorotate dehydrogenase (DHODH), from dihydro-orotate to orotate transformation, which is involved in pyridine nucleotide synthesis [59].

Further, CoQ also participates in the dissipation of energy from the ETC by the dissemination of the proton gradient as heat. CoQ associates to uncoupling proteins localized in the IMM playing in its regulation [60].

Moreover, it has recently been shown that the oxidation of ubiquinol by complex III of the ETC is an obligatory reaction to maintain tumor growth [61].

Another important aspect of the essential participation of CoQ in mitochondrial physiology is its role as a structural element in complexes I and III in the ETC [62,63]. In fact, CoQ is essential for the maintenance of the structure of complex III since the supplementation of yeast with CoQ restores the assembly of complex III in CoQ-deficient strains [64], and CoQ is also involved in complex I stability [65]. Interestingly, the deterioration of complexes I and III has been associated with the progression of different neurodegenerative diseases [66,67]. Further, defects of complexes downstream of the CoQ site end in the accumulation of ubiquinol in ETC that destabilizes complex I by ROS production, generating a vicious cycle [65].

CoQ is also a component of respirasome [68,69], and also participates in the assembly and the dynamics of supercomplexes [70]. Recently, it has been indicated that OPA1, a regulator of the fusion of the mitochondrial outer membrane, mediates the regulation of complex IV activity through a CoQ-dependent procedure [71]. Interestingly, a pool of CoQ is associated with complex I + III + IV supercomplexes, whereas free CoQ is dedicated to complex II-dependent respiratory chain activity [72,73,74]. This is very important as supercomplexes are associated with a balanced ETC chain activity and point to the regulatory role of CoQ in their assembly dynamics [70,75,76]. CoQ is also importantly implicated in the dynamics of the assembly and stability of these supercomplexes involved in mitochondrial efficiency that, when altered, induce mitochondrial dysfunction-mediated metabolic diseases and aging [77,78].

Recently, another key function of CoQ in mitochondria has been found in the outer membrane [79]. MitoNEET, also known as CDGS1 iron sulfur domain 1 (CISD1) protein, is a redox-active and pH-sensing protein that regulates energy metabolism, iron homeostasis, and ROS in mitochondria. MitoNEET interacts with reduced flavin mononucleotide (FMNH2) that reduces mitoNEET sulfoferric [2Fe–2S] clusters, which are oxidized back with CoQ being the most efficient electron acceptor [39,57]. As one of the proteins repaired by MitoNEET is the iron-master regulator IRP-1, which limits iron access to mitochondria to protect against ferroptosis in high ROS production, CoQ can be considered as an important redox-sensing factor in the adaptive response against oxidative injury, and a key component in the prevention of ferroptosis caused by mitochondrial dysfunction [39].

Finally, it has been suggested that CoQ inhibits the calcium-dependent opening of the mitochondrial permeability transition pore (PTP) and modulates the mitochondrial uncoupling proteins (UCPs) [40].

### 3.2. Extramitochondrial Redox Reactions

Since the discovery that extramitochondrial ubiquinol possesses antioxidant functions, efforts have been made to characterize the physiological enzyme reduction systems. It has been widely described that CoQ and vitamin E inhibit lipid peroxidation by scavenging lipid peroxyl radicals, and that CoQ directly scavenges the perferryl radical, thereby preventing the initiation of lipid peroxidation [57,80].

These important functions require continuous regeneration of ubiquinol from the oxidized ubiquinone and different quinone reductases have been proposed. In this way, the cytosolic enzymes that reduce extramitochondrial ubiquinone can be grouped depending on their dependence on NADPH and NADH and if they have flavin adenine dinucleotide (FAD) as prosthetic group [80]. The highest rate of extramitochondrial ubiquinone reduction described is accomplished by flavoenzymes belonging to a unique family of pyridine nucleotide oxidoreductases, such as lipoamide dehydrogenase (LipDH), mammalian thioredoxin reductase (TrxR-1), and glutathione reductase (GR) [80]. LipDH reduces CoQ with either NADH or NADPH, works at acidic pH, and its activity depends on the presence of zinc. TrxR-1 is a selenoenzyme with very broad substrate specificity that has an optimal physiological pH of 7.5 and uses either NADPH or NADH, being the most efficient ubiquinone reductase so far tested. GR is a flavoenzyme, the activity of which is also stimulated by zinc, and has an acidic pH.

On the other hand, NAD(P)H:quinone acceptor oxidoreductase1 (NQO1) plays a central role in the processes of stress adaptation, including oxidative stress [42]. NQO1 and cytochrome b_5_ reductase (CYTB5R3) regulate the stress adaptation response as a central component of the transplasma membrane redox system that is responsible for preventing lipid peroxidation by reducing oxidized antioxidants, such as vitamins E and C, and the proper CoQ [81,82]. An overexpression of these enzymes in mice improved health- and life-span by modulating lipid-mediated respiration [83,84,85]. CoQ is also involved in alterations in the redox environment by receiving highly energized electrons from NAD(P)H, and possibly in the local regulation of NAD(P)H-dependent enzymes, such as sirtuins acting as metabolic switch [1,86].

CoQ in plasma membrane contributes to prevent ferroptosis, as substrate of ferroptosis suppressor protein 1 (FSP1), acting in association to the glutathione-dependent lipid hydroperoxidase glutathione peroxidase 4 (GPX4) [87,88]. The relevant extramitochondrial redox reactions that depend on CoQ, which are integrated with other pathways, are essential to regulate cellular homeostasis and metabolism [57].

CoQ also plays an key antioxidant role in the prevention of oxidative damage in plasma cholesterol lipoproteins, essentially in low-density lipoproteins (LDLs) [89]. This role has been essentially considered in the prevention of, and protection against, cardiovascular diseases and atherogenesis [4,90]. Interestingly, a NADH-dependent reductase able to reduce LDL-associated CoQ has been found in the outer side of the plasma membrane of hepatocytes [91], possibly indicating a mechanism to maintain the redox cycle of CoQ in plasma. The importance of this enzyme in the maintenance of ubiquinol in plasma in aging and metabolic diseases is under study.

## 4. CoQ Deficiency Syndrome

CoQ deficiency syndrome includes a subgroup of very heterogeneous mitochondrial diseases characterized by reduced levels of CoQ in cells. The first cases of CoQ deficiency were described in 1989 by Ogasahara et al. [92]. This report showed two sisters displaying a progressive muscle weakness characterized by an abnormally low activity of complex I + III (NADH-cytochrome c reductase) and II + III (succinate-cytochrome c reductase). This indicates that the activities that needed CoQ were low, while the individual complex activities were normal [92]. 

Taking into consideration the essential cellular functions carried out by CoQ in mitochondria [57], a depletion of these molecules mainly leads to the impairment of OXPHOS, causing lower rates of ATP synthesis, and rising ROS generation through the disruption of its participation in supercomplex formation [65,69]. CoQ deficiency leads to the development of mitochondrial diseases affecting organs or tissues with higher CoQ levels and bioenergetic requirements. Although these processes are the main features of CoQ deficiency, other processes where CoQ is involved contribute significantly to the development of the diseases [57]. Generally, CoQ deficiency has been associated with many age-related diseases [39], neurological disorders [93], kidney and liver diseases [94,95], and heart failure [96], among others.

Several causes, including reduced dietary CoQ intake, excessive CoQ cell metabolism, and biosynthesis defects, have been proposed as being important to the development of CoQ deficiency syndrome [97,98]. To date, although the number of cases reported is increasing, the pathophysiology of this syndrome is still poorly understood. It has been suggested that several mechanisms could contribute to development of the disease, such as the pleiotropic functions of CoQ, genotypic variability, and the unknown functions of CoQ intermediates in the biosynthesis pathway [14].

### 4.1. Primary CoQ Deficiency

#### 4.1.1. Characteristics of Primary CoQ Deficiencies

Primary CoQ deficiencies are autosomal recessive conditions caused by a biallelic mutation in gene-encoding proteins implicated in the CoQ biosynthesis pathway [19]. These diseases in humans are highly heterogeneous and with a broad spectrum of clinical phenotypes. The estimated overall prevalence of this primary form is less than 1:100,000 [99]. Generally, patients manifest a combination of symptoms ranging from isolated affects on organs or tissues, such as the kidney, skeletal muscle, the heart, or the brain, to complex multisystem disorders with different severity levels and age of onset [19,97,100]. To date, defects in *PDSS1*, *PDSS2*, *COQ2*, *COQ4*, *COQ5*, *COQ6*, *COQ7*, *COQ8A*, *COQ8B*, and *COQ9* genes have been described in CoQ deficiency-related diseases. However, there is no relationship between mutation and a specific phenotype. In this regard, mutations in *COQ8A/ADCK3* cause ataxic cerebellar syndrome, and *COQ8B/ADCK4* and *COQ6* genes cause steroid-resistant nephrotic syndrome, while mutations in other *COQ* genes are more pleiotropic [19]. For this reason, the most ambitious clinical challenge is to be able to establish the correlation between genotype and phenotype to accelerate diagnosis and improve therapies [19]. 

According to the clinical symptoms, CoQ deficiency has been classified in five major groups of diseases:

(1) Encephalomyopathy with recurrent myoglobinuria, myopathy, and brain involvement [101];
(2) Severe infantile multisystemic disease characterized by encephalopathy and nephropathy [102,103];
(3) Cerebellar ataxia [104,105];
(4) Isolated myopathy [106,107];
(5) Nephropathy [100].

However, this classification can cause confusion in the difference between primary and secondary CoQ deficiencies, at least until the genetic diagnostic is completed. In fact, this classification needs to be updated due to the emerging number of patients who show overlapping clinical manifestations [108]. However, in the last few years, the use of next-generation sequencing (NGS) technologies, such as whole exome sequencing (WES) and whole genome sequencing (WGS), has helped to progress rapid diagnosis [109]. This means that clinical and biochemical techniques are useful for the rapid identification of CoQ deficiency, but only molecular diagnosis may elucidate the etiology of this syndrome. 

#### 4.1.2. Relationship of Primary CoQ Deficiency and Pathological Phenotype

In order to clarify the relationship between *COQ* mutations and phenotypes, recent revisions have been published [19,50]. Many of the mutations directly affecting members of the CoQ-synthome produce effects on the central nervous system, being *COQ4* [110], *COQ5* [111], *COQ7* [112] and *COQ9* [102], the mutated genes that generate stronger developmental delays including motor, cognitive, speech, emotional, and social and communication skill dysfunctions [113]. Further, many of the mutations found in the members of the COQ-synthome have been associated with intellectual disability characterized by impaired intellectual and adaptive functioning. Further, many mutated COQ proteins have also been involved in epilepsy, hypotonia, and the peripheral nervous system, as well as sensory organ deficiencies such as hearing loss [19]. 

Many of the mutations in COQ genes also affect kidney function. In general, renal dysfunction accompanied by altered morphological and physiological instability has been found especially in *PDSS2* [114], *COQ2* [115], *COQ6* [116], and COQ8B [117] patients. All these patients evolve to end-stage renal disease within childhood if not treated, indicating the severity of the mutations. 

Surprisingly, cardiac diseases are infrequent in primary CoQ_10_-deficient patients, despite the high importance of mitochondria in cardiac physiology. To date, the most commonly reported heart defect is hypertrophic cardiomyopathy that has been found more frequently in some *COQ4* [118] and *COQ7* [112] patients, and in a few *PDSS2* [114], *COQ2* [103], *COQ9* [102], and *COQ8B* [119] patients. Other functional heart defects include valvulopathies, cardiomegaly, septal defects, and heart hypoplasia, but no clear relationship between phenotype and mutation has been found [19,50].

Despite the importance of mitochondria in muscle physiology, muscle is relatively unaffected in these patients. In primary CoQ deficiency, isolated myopathy has been not found, although it is more common in secondary deficiency patients [100,108]. However, myopathy in COQ patients has been reported but in association with a multisystemic phenotype and not always affecting all the patients with a specific mutation [19,50]. The clearest muscle physiological defects are exercise intolerance, muscle weakness, and muscle fatigue, which are found more frequently in *COQ8* patients [120]. 

Other clinical pathologies associated with these mutations include respiratory distress and apnea [118], and respiratory failure [121] but without a clear adscription to a set of COQ mutations. Liver dysfunction has also been found in some *COQ2* patients as well as in some *COQ8* patients, but again without showing a clear pathological phenotype [19,50]. 

In general, it seems clear that neurological and renal dysfunctions in addition to hearing loss are the most common manifestations of COQ gene mutations. Other pathological manifestations can be a consequence of a different degree of sensibility to mitochondrial dysfunction depending on other factors or the equilibrium with other genes. Further research is needed, although the low number of patients with a specific mutation has made it impossible to establish a clear relationship between mutation and direct phenotypic characteristics to date. 

### 4.2. Secondary CoQ Deficiency Due to Mitochondrial Dysfunction

Secondary CoQ deficiencies were found in affected OXPHOS or non-OXPHOS processes and also in non-mitochondrial process [14,122], and are much more frequent and diverse than primary CoQ deficiency [122]. 

Table 1 includes those published cases of secondary CoQ deficiency due to mitochondrial dysfunction. The mechanisms underlying these secondary diseases are poorly understood, but they can be categorized into several groups according to the origin of CoQ deficit: (1) disorders of the OXPHOS system; (2) pathogenic variants of nuclear and mitochondrial DNA-encoded proteins; (3) defects in other enzymes upstream of OXPHOS; (4) cholesterol metabolic impairment; (5) defects in protein carriers; and (6) defects in mitochondrial homeostasis. 

The first group of these diseases is related to the essential role of CoQ in the function of ETC. Therefore, the impairment of this system promotes mitochondrial bioenergetic disorders such as defects in proteins like BSC1L, necessary for assembling CIII [123], the CI subunit NDUFSA [124,125], and the mitochondrial protease PARL, necessary for the processing of TTC19 required for CIII activity [67]. In these cases, the reduction in CoQ levels could be the cause of a negative feedback of CoQ biosynthesis by decreasing the activity of respiratory complexes. 

The maintenance of DNA copy number and its integrity is essential to preserving a proper mitochondrial function. The second diseases group is led by defective nuclear and mitochondrial-encoded OXPHOS proteins. Some of these alterations include nuclear DNA deletions [126] and mutations in *POLG*, *MPV17*, *SUCLA2*, or *FBXLA* genes [127,128], which cause mitochondrial DNA depletion syndrome (MDS) or Alper’s disease. Both mitochondrial and nuclear genome mutations can generate defects in the translation of mitochondrial proteins. Thus, mutations in *EARS2*, which encodes for mitochondrial aminoacyl-tRNA synthetase, cause mild-type leukoencephalopathy [129], and *MT-TL1* and *MT-TK* mutations generate MELAS [130] and MERRF [131] diseases, respectively.

CoQ also participates as a cofactor in other enzymatic reactions upstream and downstream of the OXPHOS system. For example, as indicated above, CoQ receives electrons from many other dehydrogenases, such as electron-transferring flavoprotein dehydrogenase (ETFDH). Mutation in this gene causes multiple acyl-CoA dehydrogenation deficiency (MADD) characterized by a decrease in CoQ levels [132]. CoQ deficiency has been found in cardiofaciocutaneous syndrome where the *BRAF* gene, encoding a serine/threonine-protein kinase, is mutated [133]. Mutations in *ACADVL*, causing very long-chain Acyl-CoA dehydrogenase deficiency, and *TBC1D24*, which causes multifocal polymyoclonus and neurodevelopmental delay, have been associated with secondary CoQ deficiency [134,135]. 

CoQ is distributed throughout the cell membranes after synthesis inside mitochondria, and extracellular CoQ can be imported into mitochondria [15,148]. In yeast, the distribution of endogenously synthesized CoQ in mitochondria is affected by the *Ypl109c* and *Ylr253w* genes. *Ypl109c* (*Cqd1*) is required to maintain CoQ in mitochondria. Its deletion increases CoQ exit from mitochondria, increasing the resistance to oxidative stress. *Ylr253w* (*Cqd2*) promotes the opposite effects [149]. Interestingly, *Adck2* (a mammal orthologue of *Ypl109c*) deletion causes mitochondrial deficiency in CoQ, leading to mitochondrial myopathy in skeletal muscle [150]. To reach mitochondria, exogenous CoQ requires a mechanism of import based on endocytosis and intracellular vesicular trafficking pathways, well conserved in all species. To date, six yeast transporter proteins (CDC10, RTS1, RVS161, RVS167, VPS1, and NAT3) represent the essential steps in the pathways responsible for the transport of exogenous CoQ to its functional sites in the cell [151]. Additionally, in yeast, the CoQ biosynthetic complex is organized in domains related to endoplasmic reticulum-mitochondria encounter structures called ER-mitochondria contact sites. Disorganization of these sites triggers secondary CoQ deficiency through a loss of mitochondrial membrane integrity compromising Q-synthome organization and the transfer of CoQ from and to mitochondria [21,152]. 

CoQ-synthome and mitochondrial machinery organization could also be affected by a disruption of intracellular cholesterol trafficking towards mitochondria. In fact, an elevated mitochondrial cholesterol level has been detected in several mitochondrial pathological conditions, including Niemann-Pick Type C1-deficiency (NPC) [141], steatohepatitis [140], and Alzheimer’s disease [139]. A study using in vitro and in vivo models of mitochondrial cholesterol enrichment displayed an impairment in oxidative phosphorylation and respiratory supercomplex assembly. This effect is due to the dysregulation of the enzyme in cholesterol synthesis 3-Hydroxy-3-Methylglutaryl-CoA Reductase (HMG-CoAR), which may down-regulate the mevalonate pathway and CoQ synthesis [139]. Furthermore, mitochondrial cholesterol trafficking is controlled by proteins such as STAR and MLN64, among others. An overexpression of MLN64 [142] and mitochondrial aquaporin-8 (mtAQP8) [143] induce an increase in both mitochondrial cholesterol and dysfunction. In fact, it has been hypothesized that statin-induced CoQ deficiency inhibits HMG-CoAR, a common enzyme of CoQ and the cholesterol biosynthesis pathways [138].

The fifth group of causes of secondary CoQ deficiency is represented by a deficit in the activity of transporter proteins. The cholesterol could perturb other membrane carriers such as SLC25A11, which catalyzes the transport of 2-oxoglutarate to mitochondria. A cholesterol-mediated impairment of this carrier promotes mitochondrial GSH (mGSH) depletion, leading to increased oxidative stress [145]. Furthermore, mutations in the *ANO10* gene, which encodes for a carrier implicated in calcium signaling that is important for mitochondrial activity, has been associated with CoQ deficiency and ataxia [146]. Mutations in the *FXN* gene encoding for frataxin, a protein that regulates the transport of iron into mitochondria, is related to ferroptosis, a regulated cell death associated with lipid peroxidation. Some patients with Friedrich ataxia harbored the *FXN* mutation associated with low CoQ levels in skeletal muscle [122]. Recently, it has been observed that CoQ performs an important role in the DHODH-mediated defense mechanism against ferroptosis [153,154]. 

Finally, mitochondrial homeostasis is the result of a balance between mitochondrial biogenesis and mitophagy. This last group of secondary CoQ deficiencies is represented by diseases in which mitochondrial dysfunction is the result of an unbalance in mitochondrial turnover. In Familiar Hypercholesterolemia (FH), pathology has been associated with the persistent activation of mitophagy in fibroblasts [144]. Similarly, an increase in the degradation of impaired mitochondria was observed in fibroblasts of patients with MELAS [130]. Furthermore, MFN1 and MFN2 are also proteins implicated in mitochondrial dynamics through the activation of fusion to increase the amount of active mitochondria [155]. Specifically, it has been described that MFN2 is essential for CoQ biosynthesis [147]. 

**Figure 2:**
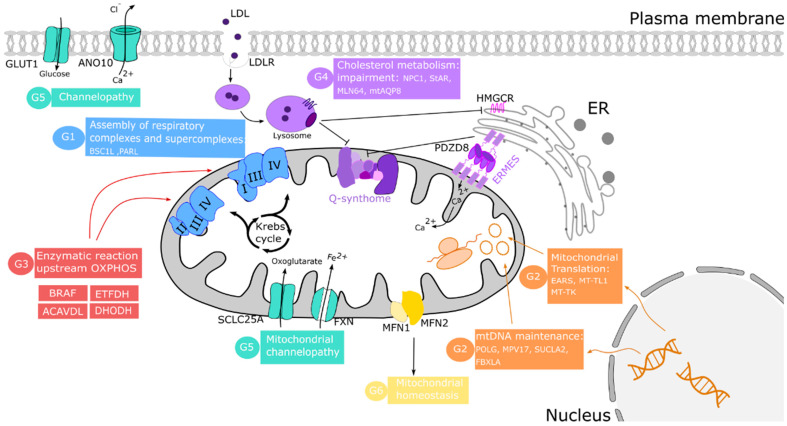
Causes of secondary CoQ deficiency. Mechanisms proposed to trigger the secondary variant are grouped in several categories (G1 to G6) indicated in different colors. The synthesis of CoQ can be reduced by defects in proteins implicated directly or indirectly in the assembly and proper respiratory machinery functioning (G1 (blue) and G3 (red)). In orange (G2) are the essential proteins for the maintenance of intact mtDNA. Point mutations in mitochondrial aminoacyl-tRNA synthetases are also associated with low CoQ levels. The stability of Q-synthome depends on ER-mitochondrial contact sites (ERMES) and the cholesterol synthesis pathway (G4 (purple)). Defects in the proteins implicated in the transport of several metabolites, such as glucose or Fe^2+^, throughout to plasm or mitochondrial membrane cause channelopathies, marked in green (G5). The mechanisms to maintain mitochondrial mass by processes such as fusion-fission proteins, MFN1, and MFN2 (G6) are shown in yellow.

In summary, CoQ synthesis can be affected by many different processes located not only in mitochondria, but also in other organelles (Figure 2). 

## 5. Role of CoQ in Aging and Age-Associated Metabolic Disorders

The accumulation of dysfunctional mitochondria is a shared feature of both aging and metabolic disorders [156,157,158,159]. Evidence indicating the essential role of mitochondrial physiology in the increasing dysfunction of tissues and organs during aging has been accumulating over the last few years [160,161]. This indicates that any therapy able to maintain a balanced mitochondrial turnover and dynamics can delay age-associated metabolic diseases and improve functionality during the progression of aging [1,4,39]. 

The essential role of CoQ in the maintenance of mitochondrial activities and in the prevention of oxidative damage in cells and plasma lipoproteins points to its importance in aging and in age-related diseases [1,162]. However, it is not clear if the decrease in CoQ levels associated with aging is the cause of mitochondrial dysfunction or a consequence of the deterioration of the turnover and dynamics of mitochondria found in metabolic diseases and aging [157,160,163].

We can consider that the secondary CoQ deficiency associated with aging may be a consequence of OXPHOS dysfunction [126]. Several CoQ biosynthesis genes were downregulated in mice showing impaired mtDNA gene expression [126]. Many components of the CoQ-synthome suffered a clear decrease in mitochondria: COQ3, COQ5, COQ6, COQ7, COQ8A/ADCK3, COQ9, and COQ10A. On the other hand, two of these enzymes (PDSS2 and COQ8B/ADCK4) increased, indicating a different response of the members of the synthome to mitochondrial dysfunction [126]. Additionally, COQ8A/ADCK3 and COQ8B/ADCK4 are regulated depending on the glycolytic or respiratory conditions, indicating a response to the metabolic conditions [164]. It has been recently reported that in glioma cells, the inhibition of CoQ biosynthesis is associated with the stabilization of HIF-1 and the switch toward glycolysis, introducing a mechanism in the regulation of the metabolism and the development of cancer [165].

The decrease in CoQ levels found in mutants and in aged animals could be a response to the equilibrium to maintain a balanced activity of the ETC. However, we cannot discard a side effect of OXPHOS dysfunction that affects activities in the inner mitochondrial membrane that regulate CoQ synthesis [67], and the transport of proteins into the mitochondrial matrix that can destabilize the CoQ-synthome [126].

Interestingly, the maintenance of balanced mitochondrial dynamics is essential to avoid mitochondrial dysfunction during aging and metabolic diseases [156]. The transport of polyprenyl pyrophosphate from endoplasmic reticulum to mitochondria is severely affected in mitofusin2 (MFN2) KO mice and causes CoQ deficiency [147]. MFN2 is directly involved in mitochondrial fusion [166], and also in the tethering of mitochondria to the ER [167]. Communication between the ER and mitochondria affects many physiological processes, and its alteration has been associated with aging [168] and many chronic pathologies associated with aging, such as neurodegenerative diseases, metabolic syndromes, and cancer [169]. 

On the other hand, a depletion in CoQ levels activates mitophagy [170] and increases the dysfunction of mitochondria associated with higher oxidative stress and apoptosis in cultured cells [171]. Further, a depletion in CoQ levels would not only add more disturbing factors for mitochondrial physiology, but also affects membrane antioxidant activities. This cycle would accelerate mitochondrial dysfunction while aggravating oxidative stress. 

In support of the key role of CoQ in the delay of this vicious cycle, recent studies have proposed the therapeutic use of CoQ_,_ or bioactive compounds able to increase its levels, to reduce the progression of age-related diseases and to improve healthy aging [39,172,173]. Treatments with ubiquinol can rescue statin-associated mitochondrial dysfunction and rhabdomyolysis, indicating that a depletion in CoQ levels in muscle causes mitochondrial dysfunction through the chronic use of statins [174]. Lower CoQ levels have been recently associated with the progress of chronic kidney diseases [175]. 

## 6. Supplementation with CoQ: Metabolic Recovery

The efficacy of CoQ as supplement for the treatment of diseases associated with mitochondrial dysfunction is not completely clear. Many studies and clinical trials have been performed in order to find the positive effects of dietary supplementation with CoQ in different metabolic and age-related diseases [176]. However, the design of the trials and the presence of an important percentage of the population showing a low incorporation of dietary CoQ has not allowed us to obtain clear therapeutic procedures for this compound [177,178]. 

The hydrophobicity of CoQ and its large molecular weight reduces the amount of nutritional CoQ that reaches blood plasma [179]. Fat-solubilized formulations are preferred to powder-based presentations as solubilization in the fat matrix permits superior bioavailability [178,179]. Recently, it has been found that CoQ transport in intestinal epithelium requires Niemann-Pick Like 1 protein, a transporter involved in the intestinal absorption of fat-soluble components such as cholesterol [180]. This is an interesting aspect as people showing low fat absorption from their diet may suffer from an insufficient incorporation of dietary CoQ. In any case, supplementation with CoQ produces null or very low toxicity and no serious side effects in doses up to 1.2 to 3 g/day [181]. This aspect is important, especially in those patients suffering CoQ deficiency syndrome, which needs supplementation with high levels of this compound. 

CoQ supplementation has shown therapeutic benefits in aging-related disorders, principally in cardiovascular and metabolic diseases in the elderly [182,183]. Studies on the effect of CoQ supplementation in human aging need to be performed rigorously, but studies in rat life-long supplementation have demonstrated the attenuation of an age-related decrease in total antioxidant activity, and also a reduction in DNA-damage in lymphocytes [184]. 

The supplementation of a Mediterranean diet with CoQ improves the metabolism of advanced glycation end products in the post-prandial period in elderly people [185], the lipid and glycemic profile in dyslipidemia individuals [186], and the glucose metabolism and oxidative damage markers in the plasma of patients with diabetic nephropathy [187]. In people suffering metabolic syndrome, CoQ supplementation may reduce serum triglyceride levels and improve lipid profiles [188].

Metabolic syndrome courses with a chronic inflammatory profile and CoQ supplementation has demonstrated the capacity to reduce this profile, possibly by improving mitochondrial activity in immune cells. In general, supplementation with CoQ can reduce the levels of plasmatic inflammatory cytokines [189], although the number of studies are limited, and in some cases, the results are conflicting [190,191,192]. The effect of supplementation with CoQ in a decrease in inflammation and lipid peroxidation in metabolic syndrome patients has also been associated with an improvement in liver function [193].

Mitochondrial dysfunction is associated with aging-dependent myalgia, and supplementation with CoQ shows some therapeutic efficacy in reducing inflammation and restoring antioxidant and mitochondrial activities [194]. Interestingly, CoQ and carotene deficiencies, accompanied by metabolic disorders, are common in patients with oral cancer. This deficiency is associated with an increase in the risk of central obesity, hyperglyceridemia, and metabolic disease [195]. 

Interestingly, CoQ_10_ appears as an efficient reducer of inflammation in people showing chronic inflammation processes, such as ulcerative colitis [196]. In fact, its use has been proposed in the modulation of autoimmune disorders accompanied by inflammatory processes [197], such as antiphospholipid syndrome [198]. We have recently proposed that supplementation with CoQ could reverse the mitochondrial dysfunction affecting immune cells such as cytotoxic T-lymphocytes and proinflammatory macrophages that are associated with immune deficiency and the inflammatory responses associated with respiratory diseases [49]. 

A reduction in proinflammatory cytokines levels through the modulation of mitochondrial dysfunction in endothelial and immune cells can also be related with a protective effect on neurodegenerative diseases. In these diseases, oxidative stress is considered a key modulator of the progression of cell dysfunction and degeneration [43]. In fact, supplementation with CoQ reduces chronic inflammation and endothelial dysfunction in patients suffering mild cognitive impairment [199]. It seems clear that inflammation can be considered a therapeutic target of CoQ supplementation for neuronal and muscular degenerative diseases [98,173]. 

The most clear therapeutic effect of supplementation with CoQ is in cardiovascular diseases [200], and lowering lipid levels in patients with coronary artery disease [201]. In fact, CoQ supplementation is considered to be a therapeutic tool to prevent cardiomyopathies, coronary artery disease, and the preservation of the myocardium [200]. In recent years, the combination of selenium and CoQ has shown positive effects in reducing cardiovascular mortality and the levels of plasma markers of cardiovascular damage [182,183,202,203,204].

Apart from an improvement in cardiovascular function, the protection of endothelial function against oxidative damage in cardiovascular disease seems to be one of the most promising activities of supplementation with CoQ as it can rapidly reach these cells from plasma [205]. CoQ can act as an antioxidant both reducing oxidative damage and improving mitochondrial activity reducing oxidative stress [206], which would improve endothelial function [207]. The endothelial dysfunction induced by dyslipidemia can be improved by CoQ supplementation through increasing NO production and reducing LDL oxidation [208]. 

Mitochondrial activity and oxidative damage are also associated with exercise. The antioxidant capacity of CoQ can be essential in the protection against oxidative damage and inflammation during intense exercise [209], and can even improve aerobic capacity in athletes during heavy training sessions or competition [210,211]. Short-term moderate CoQ supplementation can alleviate tissue damage and fatigue after strenuous exercise in runners [212]. Supplementation with CoQ has been proposed to restore moderate CoQ deficiency in athletes in order to maintain antioxidant protection and exercise performance [213], to induce pro-angiogenic effects by improving hemoglobin levels and reducing inflammatory markers [209], and to produce an ergogenic effect [214]. 

## 7. Concluding Remarks

It is clear that CoQ is an essential factor involved in many metabolic and antioxidant processes controlling energy in cells and in molecular protection against oxidation and even cell survival against ferroptosis. As essential factor for many different metabolic activities in the cells mainly involving catabolic, anabolic, and antioxidant activities, CoQ plays a pivotal role in cell and organ physiology. Its deficiency can displace the metabolism to those activities in which CoQ interaction occurs at higher affinity, whereas those enzymes that interact with CoQ at lower affinity are seriously affected. This effect can be important in mitochondria, in which CoQ shares its redox activity with catabolic and anabolic processes. 

For this reason, levels of CoQ in humans can be key in the promotion, initial phases, and development of many metabolic and degenerative diseases, and even in the proper aging process. Mitochondrial dysfunction and CoQ levels can be part of a vicious cycle in which dysfunctional mitochondria can reduce CoQ levels, enhancing mitochondrial destruction by mitophagy, and further increasing mitochondrial dysfunction. Supplementation with CoQ in many of the diseases involved in chronic metabolic diseases and aging can improve mitochondrial and metabolic activities and decrease oxidative damage, reinforcing the role of CoQ as a hinge in metabolism regulation in health and disease. 

## Figures and Tables

**Table 1:** Groups of disorders associated with secondary CoQ deficiency.

Group of Disorders	Gene	Function	Diagnosis	Clinical Phenotype Described in Humans	Refs.
G1: Disorders due to impairment of oxidative phosphorylation (OXPHOS) system	BSCL-1	Defective BSCL-1 generates a catalytically and structurally inactive complex III	Leigh syndrome	Neurological symptoms and lactic acidosis	[123]
	NDUFS4	Defects on assembly of functional complex I	Leigh syndrome	Progressive loss of mental and psychomotor regression	[124,125]
	PARL	Instability of TTC19 expression, which is required for complex III activity	Leigh-like syndrome due to impairment of CIII activity	Necrotizing encephalomyelopathy	[67]
G2: Defects on nuclear and mitochondrial DNA	nDNA and mtDNA deletions	OXPHOS dysfunction	Mitochondrial DNA depletion syndrome (MDS)	Clinically heterogeneous mitochondrial phenotypes	[126]
	POLG, MPV17, SUCLA2, FBXLA	mtDNA depletion			[127,128]
	EARS2	Defective mitochondrial aminoacyl-tRNA synthetase specific for glutamate	Combined OXPHOS deficiency	Leukoencephalopathy with high lactate	[129]
	MT-TL1	Translational defects by punctual mutations in tRNA encoded by mtDNA	MELAS disease	Encephalomyopathy and lactic acidosis	[130]
	MT-TK		MERFF disease	Clinically heterogeneous mitochondrial phenotypes	[131]
G3: Defects on other proteins involved in enzymatic reactions upstream of OXPHOS	ETFDH	Electron transfer defects from electron-transferring flavoprotein to ubiquinone	Multiple Acyl-CoA dehydrogenase deficiency (MADD)	Isolated myopathic phenotype	[132]
	BRAF	Disruption of signals controlling cell growth	Cardiofaciocutaneous syndrome (CFS)	Psychomotor development delayed, muscular hypotonia, and ataxia	[133]
	ACADVL	Defects of first step of the mitochondrial fatty acid beta-oxidation pathway	Very long-chain Acyl-CoA dehydrogenase deficiency	Encephalopathy and rhabdomyolysis	[134]
	TBC1D24	GTPase-activating protein for Rab family protein	Multifocal polymyoclorus	Multifocal polymyoclonus and neurodevelopmental delay	[135]
G4: Cholesterol metabolism impairment	PDZD8	Impairment of mitochondrial Ca^2+^ uptake	Alzheimer’s disease, Parkinson’s disease, and amyotrophic lateral sclerosis with associated frontotemporal dementia	Typical symptoms in neurodegenerative diseases	[136,137]
	3-Hydroxy-3-Methylglutaryl-CoA Rase	Defects on mevalonate pathway and CoQ synthesis	Niemann-Pick Type C1-deficiency, steatohepatitis, Alzheimer’s disease		[138,139,140,141]
	StAR, MLN64, Aquaporin-8	Cholesterol metabolism impairment			[142,143]
	LDL-receptor and other proteins	Binding defects on LDL-cholesterol to plasma membrane receptor	Familial hypercholesterolemia	Early atherosclerosis and elevated serum cholesterol concentrations	[144]
G5: Diseases caused by defects on protein carrier	SLC25A11	Disruption of transport of 2-oxoglutarate to mitochondria	Liver cancer	Hepatocellular carcinoma	[145]
	ANO10	Putative calcium-activated chloride channels defects	Autosomal recessive spinocerebellar ataxia-10	Slowly progressive ataxia and dysarthria	[146]
	FXN	Defects in transport of iron to mitochondria	Friedreich’s ataxia	Ataxia, limn incoordination, dysarthria, dysphagia, eye movement defects, and muscle weakness	[122]
G6: Mitochondrial homeostasis dysregulation	LDL-receptor and other proteins	Autophagy flux impairment	Familial hypercholesterolemia	Early atherosclerosis and elevated serum cholesterol concentration	[144]
	Point mutations in mtDNA genes encoding tRNAs	Defective protein synthesis	MELAS disease	Lactic acidosis	[130]
	MFN2	Depletion of mitochondrial CoQ and respiratory chain dysfunction	n.d.	n.d.	[147]

n.d.: not determined.
